# High rates of early treatment discontinuation in hepatitis C-infected US veterans

**DOI:** 10.1186/1756-0500-7-266

**Published:** 2014-04-24

**Authors:** Joanne LaFleur, Robert Hoop, Timothy Morgan, Scott L DuVall, Prashant Pandya, Eli Korner, Kristin Knippenberg, Candace Hayden, Richard E Nelson

**Affiliations:** 1 Pharmacotherapy Outcomes Research Center, University of Utah, 30 South 2000 East, Salt Lake City, UT 84112, USA; 2 VA Salt Lake City Health Care System IDEAS Center, 500 Foothill Blvd, GRECC 151, Salt Lake City, UT 84148, USA; 3 Genentech, 1 DNA Way, South San Francisco, CA 94080, USA; 4 Department of Hepatology, VA Long Beach Healthcare System, 5901 East 7th Street, Long Beach, CA 90822, USA; 5 Department of Medicine, Kansas City VA Medical Center, 4801 East Linwood Boulevard, Kansas City, MO 64128, USA

**Keywords:** Hepatitis C virus, Pegylated interferon, Discontinuation, Veterans

## Abstract

**Background:**

Patients with chronic hepatitis C (HCV) frequently discontinued dual therapy with pegylated interferon alfa (Peg-IFN) plus ribavirin (RBV) before reaching the recommended duration of 48 or 24 weeks for genotypes (G) 1/4 or 2/3, respectively. We quantified rates of discontinuation despite efficacy (non-LOE) versus lack of efficacy (LOE) versus discontinuation for unknown reasons in a national database of United States veterans.

**Methods:**

We identified a population-based cohort of U.S. veterans with encounters from 2004 through 2009 who had lab-confirmed HCV infection and initiated therapy with Peg-IFN plus RBV in Veterans Health Administration medical centers. Pharmacy data were used to determine therapy duration, defined as the sum of Peg-IFN days supplied. Patients “discontinued” if they failed to receive at least 44 (G1/4) or 20 weeks (G2/3) of therapy. We classified discontinuations as due to non-LOE, LOE, or unknown reasons using a classification rule based on treatment duration and laboratory confirmed response.

**Results:**

Of 321,238 diagnosed HCV patients during the evaluation period, 9.7% initiated therapy and 6.4% met all other inclusion criteria. 54.9% of patients discontinued early; of these, 41.2% discontinued due to non-LOE reasons, 12.5% discontinued for LOE reasons, and 46.3% discontinued for unknown reasons. Among non-LOE discontinuers, most (60.1%) discontinued in the first 4 weeks of therapy, which constitutes 13.6% of all treated patients.

**Conclusions:**

We observed a high proportion of early discontinuations with dual-therapy regimens in a national cohort of HCV-infected veterans. If this trend persists in the triple-therapy era, then efforts must be undertaken to improve adherence.

## Background

Until the 2011 approval of direct-acting antiretrovirals, the recommended first-line treatment for chronic hepatitis C virus (HCV) infection for genotypes 1–4 was a combination of pegylated interferon alfa (Peg-IFN) and ribavirin (RBV), for a duration of 24–48 weeks depending on genotype; treatment discontinuation at 12 weeks in patients failing to show at least a 2-log viral load reduction was advised [1]. This dual therapy was associated with a relatively high rate of treatment failure, largely due to poor adherence, early therapy discontinuation, and/or dose reduction due to adverse events [2,3].

The advent of triple therapy for genotype 1 patients appears to demonstrate improved response rates in clinical trials, [4,5] but because the new antiretrovirals are added to existing dual therapy regimens, poor adherence and early therapy discontinuation driven by adverse effects may persist in routine clinical practice. Furthermore, to our knowledge, no epidemiologic studies of the US veteran population have characterized the earliest discontinuers. In this study, we describe the duration of treatment and the frequencies, rates, and types of treatment discontinuation for dual therapy in a US veteran population, with particular attention paid to early discontinuations for reasons other than lack-of-efficacy (i.e., non-LOE discontinuations). The rate of early non-LOE discontinuations in dual therapy may carry over into triple therapy regardless of its increased effectiveness over dual therapy.

## Methods

### Design and datasets

We conducted descriptive analyses of dual-therapy patients from every geographic region of the Veterans Affairs (VA) healthcare system, comprising a national cohort. Datasets were acquired and analyzed within the VA INformatics and Computing Infrastructure (VINCI) environment and included Corporate Data Warehouse (CDW; height, weight, laboratory results, and patient care notes for liver-clinic patients), Medical SAS (inpatient and outpatient visits, including procedures and diagnosis codes), and Decision Support Systems (DSS; outpatient pharmacy data).

All relevant ethical safeguards have been met in relation to patient or subject protection. IRB approval for this study was obtained through the University of Utah’s IRB and the VA’s Office of Research and Development.

#### NLP methods

For patients with missing genotype in the structured laboratory data, natural language processing (NLP) techniques were used to mine the notes for the missing information. First, treated patients in the cohort with no genotype result in structured laboratory data were identified and liver clinic notes for those patients were extracted and randomized. Clinical experts then manually reviewed a subset of the randomized notes focusing on identifying sections of the note that typically contained genotype and on the typical patterns in which it is represented. Next, we used the identified sections and pattern types to build an NLP system using a character window surrounding the identified target words and processed a larger sample of the randomized notes, outputting the word window string and the extracted genotype. Clinical experts then compared the machine-extracted genotypes with the liver clinic notes to correct and flag any errors. This reference standard was used to correct identified errors in the NLP system. We repeated this iterative loop until acceptable measures of machine performance were reached. The NLP system was then used to process a final set of un-annotated randomized notes. To calculate the accuracy of the tool, clinical experts reviewed a subset of the genotype observations and checked them against the original notes to identify any errors. The system output was compared to the human-created reference standard and descriptive statistics for the performance of the tool were assessed. Accuracy was calculated as the number of observations correctly determined by the NLP tool divided by the total number of observations.

### Patient selection

We identified all United States (US) veterans with inpatient/outpatient encounters in 2004–2009 who had HCV diagnoses and laboratory-confirmed genotypes 1–4. Diagnosis codes from the 9^th^ revision of the International Classification of Diseases (ICD-9) were used to identify HCV. The subset that initiated dual therapy with RBV plus Peg-IFN 2a or 2b during the study period was included. Patients were considered to have received dual therapy if they received the first prescription for one drug within 30 days of the other.

The index date was the first prescription fill for Peg-IFN. To maximize the chances that patients would be treatment naïve, we excluded patients with prior Peg-IFN or RBV recorded in the VA dataset between 2002 and 2004 or those without at least 1 outpatient visit to the VA 180+ days prior to the index date, which we considered a demonstrated pattern of receiving routine care in the VA. We also excluded patients whose pharmacy data contained missing or invalid National Drug Code (NDC) numbers, such that we could not determine the quantities dispensed or days supplied of Peg-IFN, as well as patients who died before the end of their treatment duration (24 weeks for G2/3; 48 weeks for G1/4).

### Outcomes

The primary outcome was non-LOE discontinuation as determined by a discontinuation algorithm based on pharmacy treatment duration and laboratory patient response data (Table 1). Treatment duration was defined as the sum of Peg-IFN days supplied, which has been determined to be an adequate proxy for patient adherence to medication in large population studies [6]. Discontinuers were those who failed to complete at least 44 weeks (G1/4) or 20 weeks (G2/3) of therapy. Gaps in treatment longer than 60 days following the end of previous days supplied defined discontinuation. Discontinuations were further classified as “non-LOE” (those that occurred in the first 10 weeks before 12-week laboratory measures, or occurred after lab results documenting at least a 2-log decrease in viral load); “LOE” (those that occurred after week 10 and after lab results documenting less than a 2-log decrease in viral load), or “unknown” (those that occurred after week 10 but with insufficient laboratory evidence to determine a response). We also classified non-LOE discontinuation events by time to discontinuation: ≤4 weeks, 4–10 weeks, and ≥10 weeks. We used the 10-week threshold for the 12-week laboratory measurement to ensure that laboratory measures that were taken early were still included.

**Table 1:** Discontinuation reason classification rule

**Persistence condition**	**Laboratory condition**	**Classification**
Patient died before the end of their target treatment duration (20 weeks for G2/3 or 44 weeks for G1/4)	Any	*Excluded*^*a*^
Patient discontinued before week 10 (before the lower limit of the 12-week stopping rule)	Any	Non-LOE
Patient discontinued between week 10 (lower limit of the 12-week stopping rule) and week 20 for G2/3 (lower limit of “completion”) or week 22 for G1/4 (lower limit of the 24-week stopping rule)	Most recent quantitative or qualitative HCV-RNA since week 10 was “undetectable” or 2-log lower than baseline	Non-LOE
	Most recent post-baseline quantitative HCV-RNA since week 10 was NOT 2-log lower than baseline	LOE
	Insufficient lab data (i.e., in structured data or in clinic notes) for one of the above classifications	Unknown
*G1/4 only:* Patient discontinued between week 22 (lower limit of the 24-week stopping rule) and week 44 (lower limit of “completion”)	Most recent post-baseline quantitative or qualitative since week 22 HCV-RNA is “undetectable”	Non-LOE
	Most recent post-baseline quantitative or qualitative HCV-RNA since week 22 is still detectable	LOE
	Not enough lab data (i.e., in structured data or in clinic notes) for one of the above classifications	Unknown
Patient completed 20 (G2/3) or 44 (G1/4) weeks (“completion”)	Any	Completed

Patients were classified as “LOE,” “non-LOE,” or “completed” depending on the first set of persistence and laboratory conditions met. If the patients died before the end of their treatment period or met no complete set of conditions due to a lack of laboratory observations, they were excluded from further analysis.

Key: G – genotype; HCV-RNA – laboratory test for hepatitis C virus levels; LOE – lack-of efficacy.

^a^Excluded patients were not analyzed in this study and do not contribute to the final cohort. Unknown patients were analyzed in this study and do contribute to the final cohort.

### Statistical analysis

Descriptive statistics were used to characterize patient demographics. Discontinuation frequencies were reported overall and by time to discontinuation. The percentages of patients who discontinued at each time point between genotype groups (G1/4 versus G2/3) were compared using a Chi-square test.

## Results

### NLP results

A total of 66,151 liver clinic notes for 4,410 patients were processed using the NLP tool, which yielded 75,187 observations of genotype in 4,186 patients. Human annotations on a random sample of N = 1,000 revealed an accuracy of 96.8% (95% CI = 95.7% to 97.9%) for the extracted genotype. Extracted genotypes were incorporated into the structured data for determining patient study eligibility.

### Patients

**Figure 1:**
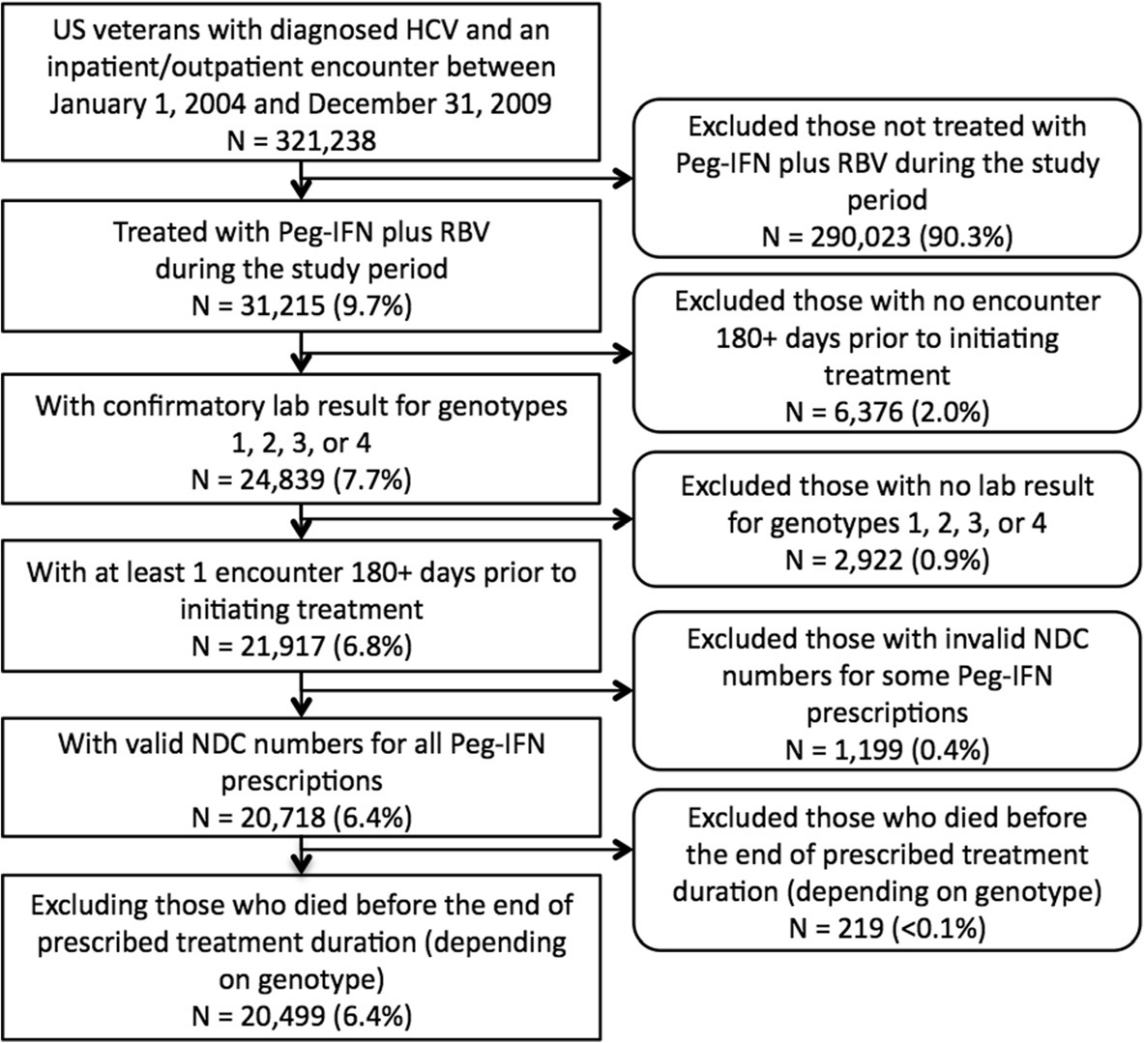
Attrition summary.

As shown in Figure 1, out of 321,238 HCV patients with an ICD-9 diagnosis code, 31,215 (9.7%) initiated dual-therapy with Peg-IFN plus RBV. Of those, we were able to identify genotype in 24,839 (79.6%) including 20,653 using structured laboratory data and 4,186 using NLP. The subset meeting all other eligibility criteria was 20,499 (6.4%; including 4,092 patients with NLP-extracted genotype), and was included in the analysis. Baseline characteristics of the cohort are summarized in Table 2. The mean (SD) age of the cohort was 53.5 (6.0), and 96.1% were male. Race was known in 42.9% of the cohort: 26.2% white, 13.4% black, and 3.3% Hispanic. The vast majority of patients were overweight (41.9%) or obese (32.7%). The most prevalent comorbidities in the cohort were tobacco use (36.6%), alcohol use (27.4%), mental health issues (24.3%), and drug abuse (24.3%).

**Table 2:** Patient characteristics, as categorized by treatment duration and discontinuation, all genotypes

	**Total (N = 20,499)**				**Discontinued for non-LOE reasons (N = 4637)**				**Discontinued due to LOE (N = 1408)**				**Unknown (N = 5202)**				**Completed ≥20/44 wks of therapy (N = 9252)**			
	**N**	**%**	**CI-LL**	**CI-UL**	**N**	**%**	**CI-LL**	**CI-UL**	**N**	**%**	**CI-LL**	**CI-UL**	**N**	**%**	**CI-LL**	**CI-UL**	**N**	**%**	**CI-LL**	**CI-UL**
**DEMOGRAPHIC VARIABLES**																				
Age																				
< 50	4636	22.6%	22.1%	23.1%	964	20.8%	19.8%	21.8%	288	20.5%	18.7%	22.2%	1074	20.6%	19.7%	21.6%	2310	25.0%	24.2%	25.7%
50-64	15388	75.1%	74.6%	75.6%	3557	76.7%	75.7%	77.7%	1085	77.1%	75.2%	78.9%	3983	76.6%	75.6%	77.5%	6763	73.1%	72.3%	73.9%
65+	475	2.3%	2.1%	2.5%	116	2.5%	2.1%	2.9%	35	2.5%	1.8%	3.2%	145	2.8%	2.4%	3.2%	179	1.9%	1.7%	2.2%
BMI																				
< 18.5	88	0.4%	0.4%	0.5%	34	0.7%	0.5%	0.9%	6	0.4%	0.1%	0.7%	16	0.3%	0.2%	0.4%	32	0.3%	0.2%	0.4%
18.5 – 24.9	4910	24.0%	23.5%	24.4%	1179	25.4%	24.4%	26.5%	316	22.4%	20.6%	24.3%	1234	23.7%	22.8%	24.7%	2181	23.6%	22.8%	24.3%
25 – 29.9	8583	41.9%	41.3%	42.4%	1941	41.9%	40.7%	43.1%	620	44.0%	41.9%	46.2%	2162	41.6%	40.4%	42.7%	3860	41.7%	40.9%	42.6%
30+	6708	32.7%	32.2%	33.3%	1423	30.7%	29.6%	31.8%	456	32.4%	30.3%	34.4%	1736	33.4%	32.3%	34.4%	3093	33.4%	32.6%	34.2%
Unknown	210	1.0%	0.9%	1.1%	60	1.3%	1.0%	1.6%	10	0.7%	0.3%	1.1%	54	1.0%	0.8%	1.3%	86	0.9%	0.8%	1.1%
Marital status																				
Married	6985	34.1%	33.5%	34.6%	1459	31.5%	30.3%	32.6%	501	35.6%	33.5%	37.7%	1784	34.3%	33.2%	35.4%	3241	35.0%	34.2%	35.8%
Not married	3480	17.0%	16.5%	17.4%	798	17.2%	16.3%	18.1%	259	18.4%	16.7%	20.1%	821	15.8%	15.0%	16.6%	1602	17.3%	16.7%	18.0%
Other/unknown	10034	48.9%	48.4%	49.5%	2380	51.3%	50.1%	52.5%	648	46.0%	43.8%	48.2%	2597	49.9%	48.8%	51.1%	4409	47.7%	46.8%	48.5%
Race																				
White	5375	26.2%	25.7%	26.7%	1184	25.5%	24.5%	26.6%	313	22.2%	20.4%	24.1%	1243	23.9%	22.9%	24.9%	2635	28.5%	27.7%	29.3%
Black	2738	13.4%	13.0%	13.7%	741	16.0%	15.1%	16.9%	274	19.5%	17.7%	21.2%	873	16.8%	15.9%	17.6%	850	9.2%	8.7%	9.7%
Hispanic	679	3.3%	3.1%	3.5%	149	3.2%	2.8%	3.6%	48	3.4%	2.6%	4.2%	162	3.1%	2.7%	3.5%	320	3.5%	3.1%	3.8%
Other/unknown	11706	57.1%	56.5%	57.7%	2563	55.3%	54.1%	56.5%	773	54.9%	52.7%	57.1%	2923	56.2%	55.1%	57.3%	5447	58.9%	58.0%	59.7%
Sex (male)	19708	96.1%	95.9%	96.4%	4484	96.7%	96.3%	97.1%	1359	96.5%	95.7%	97.3%	5011	96.3%	95.9%	96.8%	8854	95.7%	95.4%	96.0%
**LABORATORY VARIABLES**																				
Genotype																				
G1/4	14,893	100%	NA	NA	3562	23.9%	NA	NA	1405	9.4%	NA	NA	4560	30.6%	NA	NA	5366	36.0%	NA	NA
G2/3	5606	100%	NA	NA	1075	19.2%	NA	NA	3	0.1%	NA	NA	642	11.5%	NA	NA	3886	69.3%	NA	NA
Albumin																				
< 3.5 mg/dL	1139	5.6%	5.3%	5.8%	312	6.7%	6.1%	7.3%	90	6.4%	5.3%	7.5%	354	6.8%	6.2%	7.4%	383	4.1%	3.8%	4.5%
3.5+ mg/dL (normal)	17966	87.6%	87.3%	88.0%	3973	85.7%	84.8%	86.5%	1249	88.7%	87.3%	90.1%	4486	86.2%	85.5%	87.0%	8258	89.3%	88.7%	89.8%
Missing	1394	6.8%	6.5%	7.1%	352	7.6%	7.0%	8.2%	69	4.9%	4.0%	5.8%	362	7.0%	6.4%	7.5%	611	6.6%	6.2%	7.0%
ALT																				
< 30 IU/mL (normal)	1264	6.2%	5.9%	6.4%	292	6.3%	5.7%	6.9%	65	4.6%	3.7%	5.5%	306	5.9%	5.3%	6.4%	601	6.5%	6.1%	6.9%
30-90 IU/mL (elevated 1x-3x ULN)	10115	49.3%	48.8%	49.9%	2306	49.7%	48.5%	50.9%	750	53.3%	51.1%	55.5%	2670	51.3%	50.2%	52.5%	4389	47.4%	46.6%	48.3%
90+ IU/mL (3+ x ULN)	7788	38.0%	37.4%	38.5%	1726	37.2%	36.1%	38.4%	530	37.6%	35.5%	39.8%	1857	35.7%	34.6%	36.8%	3675	39.7%	38.9%	40.6%
Missing	1332	6.5%	6.2%	6.8%	313	6.8%	6.1%	7.4%	63	4.5%	3.6%	5.4%	369	7.1%	6.5%	7.7%	587	6.3%	5.9%	6.8%
Bilirubin																				
< 1.5 mg/dL (normal)	17327	84.5%	84.1%	84.9%	3846	82.9%	82.0%	83.9%	1220	86.6%	85.2%	88.1%	4332	83.3%	82.4%	84.1%	7929	85.7%	85.1%	86.3%
1.5 + mg/dL	1472	7.2%	6.9%	7.5%	387	8.3%	7.7%	9.0%	106	7.5%	6.4%	8.7%	402	7.7%	7.1%	8.3%	577	6.2%	5.8%	6.7%
Missing	1700	8.3%	8.0%	8.6%	404	8.7%	8.0%	9.4%	82	5.8%	4.8%	6.9%	468	9.0%	8.3%	9.6%	746	8.1%	7.6%	8.5%
Creatinine																				
< 1.1 mg/dL (normal)	2474	12.1%	11.7%	12.4%	537	11.6%	10.8%	12.4%	219	15.6%	14.0%	17.1%	611	11.7%	11.0%	12.5%	1107	12.0%	11.4%	12.5%
1.1 + mg/dL	2062	10.1%	9.7%	10.4%	505	10.9%	10.1%	11.6%	147	10.4%	9.1%	11.8%	529	10.2%	9.5%	10.9%	881	9.5%	9.0%	10.0%
Missing	15963	77.9%	77.4%	78.3%	3595	77.5%	76.5%	78.5%	1042	74.0%	72.1%	75.9%	4062	78.1%	77.1%	79.0%	7264	78.5%	77.8%	79.2%
Hemoglobin																				
Anemia^*c*^	754	3.7%	3.5%	3.9%	196	4.2%	3.7%	4.7%	46	3.3%	2.5%	4.0%	242	4.7%	4.2%	5.1%	270	2.9%	2.6%	3.2%
13+ g/dL m.; 12+ g/dL f. (normal)	18425	89.9%	89.5%	90.2%	4090	88.2%	87.4%	89.0%	1288	91.5%	90.3%	92.7%	4617	88.8%	88.0%	89.5%	8430	91.1%	90.6%	91.6%
Missing	1320	6.4%	6.2%	6.7%	351	7.6%	6.9%	8.2%	74	5.3%	4.3%	6.2%	343	6.6%	6.0%	7.2%	552	6.0%	5.6%	6.4%
LDL																				
< 100 mg/dL (normal)	6745	32.9%	32.4%	33.4%	1574	33.9%	32.8%	35.1%	537	38.1%	36.0%	40.3%	1847	35.5%	34.4%	36.6%	2787	30.1%	29.3%	30.9%
100 + mg/dL	9811	47.9%	47.3%	48.4%	2195	47.3%	46.1%	48.5%	636	45.2%	43.0%	47.4%	2332	44.8%	43.7%	46.0%	4648	50.2%	49.4%	51.1%
Missing	3943	19.2%	18.8%	19.7%	868	18.7%	17.8%	19.7%	235	16.7%	15.1%	18.3%	1023	19.7%	18.8%	20.6%	1817	19.6%	19.0%	20.3%
Platelet																				
< 100/mm^3^	865	4.2%	4.0%	4.5%	255	5.5%	4.9%	6.1%	69	4.9%	4.0%	5.8%	236	4.5%	4.1%	5.0%	305	3.3%	3.0%	3.6%
100 +/mm^3^ (normal)	18202	88.8%	88.4%	89.2%	4023	86.8%	85.9%	87.6%	1259	89.4%	88.1%	90.8%	4583	88.1%	87.4%	88.8%	8337	90.1%	89.6%	90.6%
Missing	1432	7.0%	6.7%	7.3%	359	7.7%	7.1%	8.4%	80	5.7%	4.7%	6.7%	383	7.4%	6.8%	8.0%	610	6.6%	6.2%	7.0%

^c^Anemia defined as hemoglobin <12 g/dL if female or <13 g/dL if male.

Key: CI – 95% confidence interval; LL – lower limit; UL – upper limit; NA – Not applicable; D/C – discontinuation; LOE – lack of efficacy; (ref) – reference value; BMI - body mass index; VISN – Veterans Integrated Service Network; COPD - chronic obstructive pulmonary disease; HIV - human immunodeficiency virus; ALT - alanine aminotransferase; ULN – upper limit of normal; LDL - low density lipoprotein.

### Discontinuations

**Figure 2:**
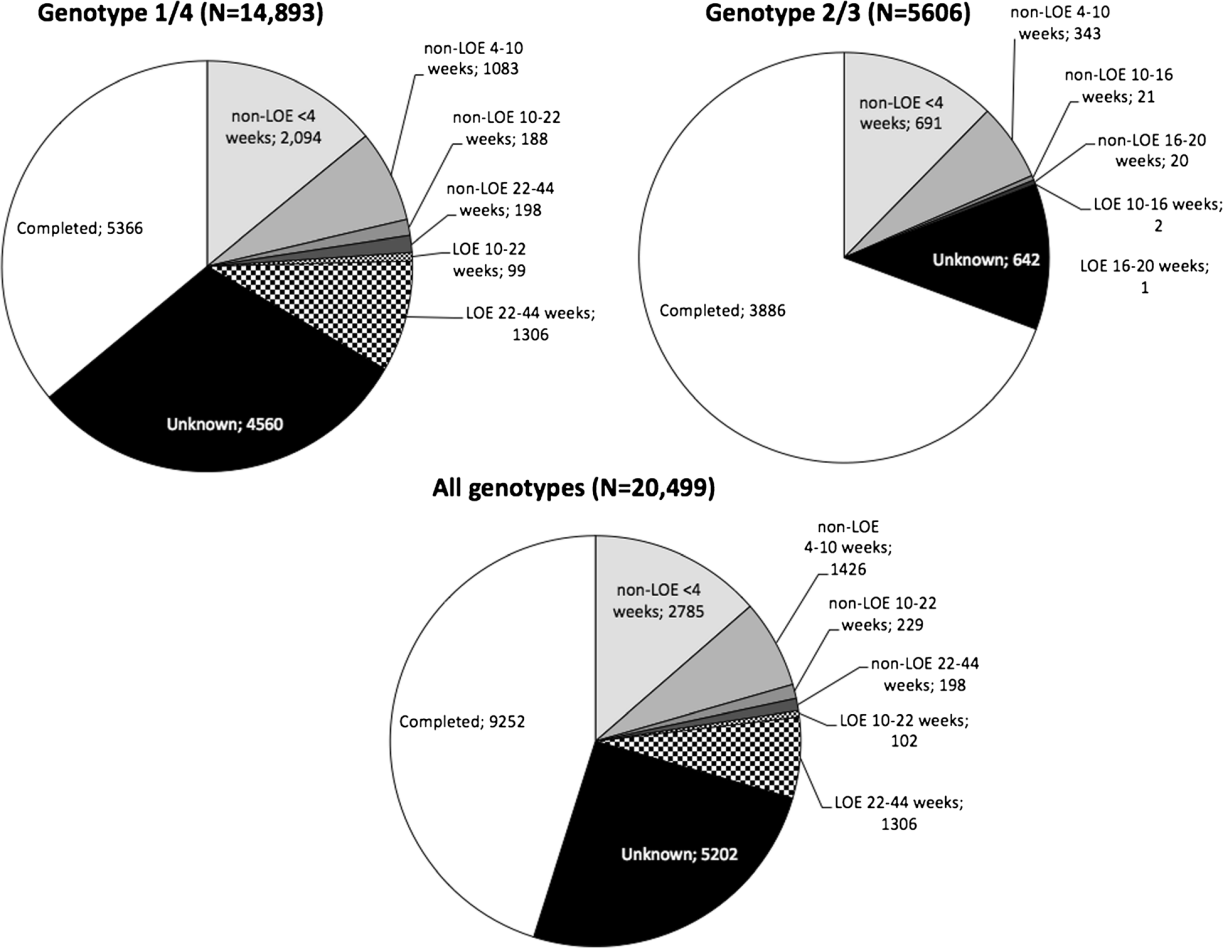
Summary of discontinuations by genotype.

As shown in Figure 2, out of the 20,499 patients who initiated treatment, over half (54.9%) failed to complete at least 44 (G1/4) or 20 (G2/3) weeks of therapy. By genotype, a much higher percentage of patients with G1/4 failed to complete therapy (64.0%) compared to G2/3 (30.7%). Our discontinuation algorithm enabled us to infer the reason for discontinuation for 53.7% of non-completing patients. Due to missing laboratory data (i.e., neither in structured data nor in clinic notes), the reasons for discontinuation could not be inferred for 25.4% of patients overall, 30.6% of patients with G1/4, and 11.5% of patients with G2/3. Among discontinuers, 41.2%, 12.5%, and 46.3% of patients were classified “non-LOE”, “LOE”, and “unknown”, respectively. By genotype, these numbers were 37.4%, 14.7%, and 47.9% for G1/4 and 62.5%, 0.2%, and 37.3% for G2/3 (see Table 2). Very few G2/3 patients were classified as LOE discontinuers.

Among the 4,637 non-LOE discontinuers, 2,785 (60.1%) discontinued in the first 4 weeks of therapy, which constitutes 13.6% of all treated patients (see Figure 2). This finding was consistent across genotype groups (58.8% of G1/4 non-LOE discontinuers and 64.3% of G2/3 non-LOE discontinuers). Discontinuation for non-LOE reasons occurred less after the initial 4 weeks of treatment, but the vast majority still discontinued before 10 weeks. The rates of non-LOE discontinuation between 4 and 10 weeks of therapy were 30.8% overall, 30.4% for G1/4, and 31.9% for G2/3. The proportions of patients who discontinued for non-LOE reasons after a documented 2-log lowering in viral load beyond week 10 were 9.2% overall, 10.8% for G1/4, and 3.8% for G2/3. Differences in the proportions who discontinued at each time point were statistically significantly for G1/4 versus G2/3 (*p* < 0.01).

## Discussion

We have documented dual therapy discontinuation rates for HCV patients in a national cohort of US veterans and estimated the percentages that discontinued for LOE and non-LOE reasons. We found that, while 69.3% of patients with G2/3 completed therapy, only 36.0% of patients with G1/4 did so. Furthermore, of those who could be classified as non-LOE discontinuers, 90.9% discontinued before the first follow-up lab test typically performed at 10–12 weeks, and 59.5% discontinued within the first month.

The high, early, non-LOE discontinuation rates we observed are very concerning. We theorize that poor tolerability of the regimen is a likely explanation. A recent survey of managed-care patients found that 69% of patients listed physical side effects and 36% listed mental side effects as the most difficult aspects of dual therapy [7]. The addition of protease inhibitors to the regimen will likely do nothing to mitigate the adverse effect profile of HCV treatment, and may worsen it.

These findings have important implications for the new triple therapy regimens. While clinical trials have shown the new protease inhibitors substantially improve treatment efficacy, [4,5] early discontinuations could limit the potential effectiveness of these regimens in clinical practice. Treatment algorithms for boceprevir-based triple therapy regimens recommend a 4-week lead-in phase with dual therapy before adding the protease inhibitor; [8] our findings suggest that a large proportion of patients may discontinue in the first month, and hence would not continue to the boceprevir combination phase. For telaprevir-based regimens, guidelines recommend the initiation of the protease inhibitor on day 1 along with Peg-IFN and RBV [8]. In this case, if the high early discontinuation rates observed with dual therapy persist in the context of triple therapy, then the cost of a month or more of a protease inhibitor will simply be added to a failed attempt at treatment in a large proportion of veterans. This suggests that adherence-enhancing initiatives will be crucial for ensuring that the efficacy benefits of protease inhibitors can be translated from clinical trials to the real-world setting.

The superior completion rates and the lower rate of unknowns among patients with G2/3 compared to G1/4 are most likely explained by two factors. First, the treatment duration for G2/3 is shorter than for G1/4 (24 weeks vs. 48 weeks), and in our study, patients with G2/3 had to persist only 20 weeks, compared to 44 weeks for G1/4. However, even at 4 weeks, the discontinuation rate was higher for patients with G1/4 compared to G2/3 (14.1% vs. 12.3%; p-value <0.001). The second explanation has to do with the response rates of the different genotypes. Since patients with G2/3 are more likely to have a rapid virological response (RVR, defined as undetectable HCV RNA at week 4 of treatment; 70-75% of G2/3 achieve RVR) compared to genotype 1 (15-25% achieve RVR), [1,9,10] we theorize that earlier positive feedback in the form of RVR may encourage patients to persist. We were nonetheless surprised to find that merely three G2/3 patients discontinued due to LOE.

To put our findings in the context of other research, only one other study has evaluated discontinuation rates in a national cohort of HCV-infected US veterans [11]. In their evaluation, Beste and colleagues focused only on genotype 1 and reported that only 30.9% discontinued early (defined as receiving refills for less than 80% of the expected treatment duration) despite evidence of a response to treatment. However, their study included only patients who had received at least 2 fills of Peg-IFN, and consequently most early discontinuers were not considered. Ours is the first study to characterize discontinuation rates for all veterans with genotypes 1–4 who received at least an initial fill of Peg-IFN + RBV.

Our study’s discontinuation rate also falls within the range cumulatively established by studies of other populations of U.S. HCV patients. Estimates of early discontinuation rates include 24% among methadone-maintained patients in California [12], 36% within a national managed care health plan [13], and 59% among a non-randomized sample of African Americans and Hispanic patients in New York [14]. Discontinuation rates also vary in U.S. veteran populations. In a regional cohort of veterans undergoing substance abuse treatment, early discontinuation estimates were 60% among G1/4 patients and 27-32% among G2/3 patients [15]. In two U.S. cohorts of primarily genotype 1-infected veterans treated with interferon or Peg-IFN, more than 75% failed to complete 48 weeks of treatment [16,17], though discontinuers are not distinguished by their reason for discontinuation. Backus et al. report cumulative days’ supply instead of weeks on treatment for genotype 1-, 2-, and 3-infected veterans; 43% of genotype 1 patients, 30% of genotype 2 patients, and 25% of genotype 3 patients did not have 100% cumulative days’ supply [18].

Although we did not collect data on SVR rates for our study, our 36.0% and 69.3% therapy completion rates for G1/4 and G2/3, respectively, are also not inconsistent with the VA SVR rates reported by Backus et al. [18]. They found SVR rates of 35%, 72%, and 62% for genotypes 1, 2, and 3, respectively. However, they only included patients who had a “post-treatment HCV RNA test”, which would be a subset of all early discontinuers. This might inflate both the proportions of therapy completion as well as SVR because of the smaller denominator.

### Limitations

Our study is subject to several limitations. First, although we carefully tracked medication possession through pharmacy refill records, as is commonly done in large-scale, epidemiologic database studies, we have no way of knowing how many doses were actually taken. Pharmacy refill records are widely accepted as a proxy for adherence in such situations [6]. Another significant limitation of our study concerns missing data: not only the high proportion of patients missing demographic data such as race (57.1% of our cohort), but especially patients with unknown reasons for discontinuation (i.e., patients with insufficient laboratory data for assessing response at week 12). We were unable to classify discontinuation outcomes for 30% of our cohort, or 46% of all discontinuers. The implications of this limitation depend on the underlying causes of the missing observations. If patients with missing laboratory response data (i.e., neither in structured data nor in clinic notes) were mostly responders, then the proportion of non-LOE discontinuers after week 10 could be much higher than we estimate here. If patients with missing laboratory response data were mostly non-responders, then we would have a much higher percentage of LOE discontinuers. However, there would be no difference in the proportions of early discontinuers reported here. A final limitation is our lack of data on the specific non-LOE reasons for treatment discontinuation in those patients, whether side effects, inconvenience, or other reasons. It is possible that a proportion of non-LOE discontinuers would have the reason recorded in their clinical notes; future work may include developing an NLP tool to extract reasons for non-LOE discontinuation.

Our decision to include the “acute hepatitis C infection” diagnosis code as an inclusion criterion may have been controversial. In fact, almost one-third of our cohort had at least one diagnosis code for acute infection. We arrived at the decision to include them because clinician experts on our research team felt that acute hepatitis C is very rare and that most patients with this diagnosis were likely misclassified; this conclusion is also supported by research showing that most acute infections are asymptomatic and are thus undiagnosed [19]. In addition, while there is little consensus on how to approach treatment of acute infections, most recommend a 4–6 month waiting period to see if the patient spontaneously clears the virus. After that, among those who fail to clear the virus, treatment options range from a course of monotherapy with interferon [20] to treatment with the full 24- or 48-weeks of dual therapy. Those treated with monotherapy would have been excluded (since a second inclusion criterion was that they had to have initiated dual therapy) while those treated with dual therapy would have been representative of the discontinuation rate we were trying to characterize, and thus it was legitimate to include them. Thus, our analysis is robust to this concern.

## Conclusion

We observed a high proportion of early discontinuations with dual-therapy regimens in a national cohort of HCV-infected veterans. If this trend persists in the triple-therapy era, then efforts must be undertaken to improve adherence so that the efficacy benefits of the new protease inhibitors can be realized by patients.

## Competing interests

This work was sponsored by a grant from Genentech. Co-authors Robert Hoop and Eli Korner are employees of Genentech and own Genentech stock. Dr. LaFleur has also been partially supported by Agency for Healthcare Research and Quality (AHRQ; HS018582) during the writing of this manuscript and has received research grants from Amgen, Genentech, Merck, Novartis, and Anolinx. Dr. Morgan has received research funding to perform clinical trials from Genentech, Hoffmann-LaRoche, Merck, Bristol-Myers-Squibb, Gilead and Vertex. Dr. Pandya has received research funding from Genentech, Hoffmann LaRoche, and Merck. Dr. DuVall has received research grants from Anolinx, LLC, Genentech, Roche, Amgen, and Shire, and was a Partner in 2010–2011 for Clinical Methods. Ms. Knippenberg and Ms. Hayden are University of Utah employees but have no other competing interests to declare. Dr. Nelson was supported in part by funding from the National Institutes of Health and the National Cancer Institute grant 1 KM1CA156723 and the National Institutes of Health Office of the Director grant 5TL1RR025762-03. No other competing interests are declared.

## Authors’ contributions

JL: took the lead in planning/conducting the study, analyzing and interpreting the data, and drafting the manuscript. She has approved the final draft submitted. RH: assisted in planning the study, interpreting the results, and contributed to drafts of the manuscript. He has approved the final draft submitted. TM: assisted in planning the study and interpreting the results, and contributed edits to drafts of the manuscript. He has approved the final draft submitted. PP: assisted in planning the study and interpreting the results, and contributed edits to drafts of the manuscript. He has approved the final draft submitted. EK: assisted in interpreting the results and contributed edits to drafts of the manuscript. He has approved the final draft submitted. KK: contributed substantially to the writing and editing of the manuscript. She has approved the final draft submitted. CH: contributed to analyzing the data and writing/editing the manuscript. She has approved the final draft submitted. SD: oversaw the Natural Programming Language (NPL) component of this study and contributed to drafts of the manuscript. He has approved the final draft submitted. RN: contributed substantially to planning the study, analyzing the data, interpreting results, and drafting/editing the manuscript. He has approved the final draft submitted. JL is the author accepting full responsibility for the conduct of the study. She has had access to the data and control of the decision to publish. All authors read and approved the final manuscript.
